# Visibly constraining an agent modulates observers’ automatic false-belief tracking

**DOI:** 10.1038/s41598-020-68240-7

**Published:** 2020-07-09

**Authors:** Jason Low, Katheryn Edwards, Stephen A. Butterfill

**Affiliations:** 1 School of Psychology, Victoria University of Wellington, Wellington, 6140 New Zealand; 2 School of Sport, Health and Wellbeing, Plymouth Marjon University, Plymouth, PL6 8BH UK; 3 Department of Philosophy, University of Warwick, Warwick, CV4 7AL UK

**Keywords:** Psychology, Human behaviour

## Abstract

Our motor system can generate representations which carry information about the goals of another agent’s actions. However, it is not known whether motor representations play a deeper role in social understanding, and, in particular, whether they enable tracking others’ beliefs. Here we show that, for adult observers, reliably manifesting an ability to track another’s false belief critically depends on representing the agent’s potential actions motorically. One signature of motor representations is that they can be disrupted by constraints on an observed agent’s action capacities. We therefore used a ‘mummification’ technique to manipulate whether the agent in a visual ball-detection task was free to act or whether he was visibly constrained from acting. Adults’ reaction times reliably reflected the agent’s beliefs only when the agent was free to act on the ball and not when the agent was visibly constrained from acting. Furthermore, it was the agent’s constrained action capabilities, rather than any perceptual novelty, that determined whether adult observers’ reaction times reliably reflected the agent’s beliefs. These findings signal that our motor system may underpin more of social cognition than previously imagined, and, in particular, that motor representations may underpin automatic false-belief tracking.

## Introduction

Tracking what is likely to happen in dynamic social situations is challenging, and—because observing others activates our own motor systems—one view is that motor representations facilitate action and social understanding. Motor representations are characteristically involved in control of small-scale actions such as dipping a brush into a can of paint, cracking an egg or placing a book on a shelf. Among other things, they enable the early parts of an action to anticipate future parts, as when you grasp a book awkwardly in order to be able to put it on a high shelf^1–3^. Research suggests that the very motor representations responsible for action control also play a role in facilitating social understanding^4^. To date, evidence for this role concerns tracking the goals of another’s action only rather than tracking mental states such as beliefs. Information about beliefs can feed into motor predictions concerning an agent’s potential actions. If this is right, motor representations might play a role in tracking not only action goals but also beliefs.

Converging evidence revealed through brain imaging, transcranial magnetic stimulation (TMS), and reaction times suggests that observation of an agent’s action leads to activation of a corresponding motor representation in the observer^4^. Neuroimaging studies support that the observation of actions done by others triggers activity in the motor execution-related brain areas^5,6^. Consistent with imaging data, studies applying TMS to the motor cortex during observation of reach-to-grasp actions reveal increased excitability in those parts of the motor cortex that correspond to the observed movement^7,8^. Most importantly for the present study, behavioural studies profiling reaction-time benefits on motor priming tasks indicate that movement observation (a task-irrelevant stimulus showing an agent lifting her index finger, for instance) can accelerate our execution of the same action and decelerate our execution of an incompatible action^9,10^.

Tracking others’ behaviour involves being sensitive not just to an action itself but also the context in which specific actions are embedded. Iacoboni et al.^11^ monitored adults’ brain activations as participants watched three types of movies: clips of background contexts (cup, teapot and plate of food arranged as if someone was about to have breakfast, or had finished); clips of a hand physically executing different grips (precision or whole-hand) to grasp a cup without context; and clips of a hand physically executing different grips to grasp a cup in the different contexts. The imaging data showed that the condition where participants witnessed grasping actions in embedded contexts, compared to the other conditions, elicited higher cortical motor activations. Motor representations can code more complex goal outcomes (grasp for drinking, grasp for putting away) of which the immediate action is a part.

Motor representations can also carry information about the features of a situation even in the absence of any movement towards a target object. Costantini et al.^12^ invited participants to execute a grip with either their left or right hand upon the presentation of a task-irrelevant go-signal (a virtual mug with its handle aligned to the left or right). The researchers found that elicitation of a spatial alignment effect—faster responding when the required hand to execute the motor act was compatible with the orientation of the mug handle—depended on the apparent possibility for participants to interact with the virtual object. The effect was elicited when the virtual mug appeared in near reachable space where it could be apparently acted upon by participants. In a follow-up study, Costantini et al.^13^ found that whether a virtual mug appears within a bystander’s reaching could also modulate participants’ own motor behaviour. The upshot is that motor activation can even occur without any actions being observed. Just anticipating someone else’s actions is sufficient to influence motor planning for your own actions in ways that can have measurable effects on task performance^14^.

As Costantini and colleagues’ findings^13^ that graspable objects potentially ready to an agent’s hand prime execution of participants’ own hand movements suggest, some motor representations are relatively effector specific. That said, Costantini and colleagues remind readers that there can be different levels of motor coding during action observation. Whilst some motor representations can map the low-level parameters that compose action (patterns of joint displacements or muscle activations)^15^, others can selectively carry high-level information relating to the goal of an agent’s action (i.e., the anticipated or observed outcomes to which another’s purposive behaviour may be directed)^16^. There are TMS data indicating that motor representations code the goal of an agent’s tool-mediated behaviour and not necessarily the muscular pattern required to perform the movement, and that virtual lesions to the ventral premotor cortex impair judgments about the outcomes that bodily actions are directed to but preserves judgements about which body parts are being observed^17^. Likewise, when we witness an agent perform an action goal using a *non*-typical effector (kicking a ball with her finger or grasping a pencil with her foot), there is motor facilitation in the effector muscles that we typically use to achieve that action goal (i.e., leg or hand, respectively)^18,19^. Similarly, there is evidence showing that adults, after being trained to use their feet to grasp a ball, started to respond more as if they would typically use their feet to operate with other objects such as hammers and cups, which typically involve hand movements^20^. Overall, the context of an action may be coded at multiple levels, whereby some motor representations that support social understanding may be relatively abstract for tracking the goal—and perhaps even the belief-informed goal—that an agent’s potential or overt action is directed to^4,21^.

If tracking the outcome-directedness of potential actions involves motor processing, then constraining an agent’s opportunity to act should significantly disrupt motor representations concerning another’s actions. Liepelt et al.^22^ instructed participants to lift their index or middle finger in response to a number stimulus presented between the index and middle finger of a photograph of an agent’s static hand. There was a slowing of participants’ reaction time in the condition where the observed agent’s corresponding index and middle fingers were tied to the table with metal clamps as compared to the conditions where the agent’s fingers were unrestrained or where the agent’s non-corresponding fingers (thumb and ring finger) were restrained. The fact that another person’s restraint leads to a finger-specific slowing of reaction time in the observer, even when the restraint was response irrelevant, suggests that our motor system automatically takes on the task-relevant circumstances governing someone else’s potentially upcoming goal-directed action. Similarly, Costantini et al.^13^ found that priming of participants’ own motor system to react more quickly (as demonstrated with the spatial alignment effect paradigm) was obliterated when the agent’s apparent possibility to interact with an object was temporarily restricted (a transparent panel was placed between the computer avatar and the handled mug). Overall, the physical body of an agent is constitutively relevant to certain kinds of cognition.

Extant evidence that motor representations enable us to track the goals of others’ actions, although broad, does not entail that motor representations underpin the primary ways in which human beings engage in social cognition to play a role in the tracking of beliefs which specify someone’s reasons for action^16^. Going beyond this evidence, it is possible that information about beliefs can feed into motor predictions concerning an agent’s potential actions. Indeed, in many social situations, successfully tracking an agent’s goal depends on tracking the correctness of the agent’s belief about that object^23^. Imagine that Maxine’s goal, as motorically expressed by the grasping shape of her hand, is to retrieve her coffee mug from a curtained windowsill. She mistakenly believes that the mug is behind the curtain to the left, though we know that it is hidden by the curtain to the right. If we attend solely to Maxine’s grasp, and ignore Maxine’s false belief, we will erroneously predict that she will reach past the left-hand curtain to retrieve her mug (from its actual location). On the other hand, by tracking Maxine’s belief we can generate a successful action prediction – that she will reach (with disappointment) behind the left-hand curtain. From this example, we see the challenge for action observation: if we are to track how an agent’s action will unfold, we cannot always rely on how things are but must also track her beliefs. It is clearly useful, therefore, that belief-tracking processes can influence those motor processes which underpin tracking others’ action goals. But could these motor processes also play a role in tracking others’ beliefs? Our study is the first to test the hypothesis that motor representations matter for successfully manifesting an ability to track others’ false beliefs.

The hypothesis is indirectly supported by studies showing that there are overlapping brain activations when adults perform mentalising and motor-priming tasks^24^. Training on a motor task that makes salient differences between one’s own and someone else’s motor action can also transfer to improvements in spontaneously tracking another’s visual perspective on a theory-of-mind (ToM) task^25^. These findings raise the question of whether impairing abilities to represent actions motorically might impair performance on a ToM task. To answer this question, our research experiment aims to test whether, and to what extent, visibly constraining an agent from potentially interacting with an object modulates observers’ abilities to track that agent’s belief.

In devising a task, we took inspiration from Bardi and Brass’s^26^ suggestion that a functional connection between control of motor representations and belief tracking might be studied in implicit ToM tasks where “participants’ performance depends on concurrent activations of different representations of the environment” (p.162). The suggestion is bolstered by research showing that motor processes may be important not only for identifying movement characteristics but also for tracking the social cognitions behind them^27–29^. To follow Bardi and Brass’s suggestion that motor processes may be important for tracking beliefs, we adapted Kovács and colleagues’ ball-detection task^30^, which yields a critical effect showing that adults’ rapid and automatic tracking of a bystander agent’s belief has an impact on observers’ own actions. The ball-detection task involves adults observing a ball rolling behind a wall on a table and then rolling from behind the wall to off the table. The video sequence also includes an agent who was present for some of the ball’s movements but not others and, consequently, could have a true or a false belief about the ball’s location. In the outcome phase, the agent returns, the wall is lowered, and participants must use their hand to press a button as fast as possible if the ball is present behind the wall. The task is considered to target automatic belief tracking because no reference is made to the agent’s belief about the ball’s location. The critical finding, which has been well-documented^30–36^, is that, compared to a baseline situation in which neither the participant nor agent expected the ball to be present (P−A−), participants are faster to respond when only the agent expected the ball to be present (P−A+), implying that the agent’s belief regarding the ball’s location is automatically encoded. That is, there is a task-irrelevant effect of the agent’s belief on the participant’s performance. What explains the effect of belief on response times? As already discussed, observing an agent act can make the observer herself prepared to act^7^. This might seem irrelevant. After all, participants in the ball-detection task do not see the agent act during the outcome phase of the experiment that is critical for belief-tracking. But, as we also mentioned above, this is not essential: even observing an agent who has the potential to act can trigger observers’ readiness to map the motor potentialities of someone else’s situated body onto their own representations of the environment^13,37–39^. It is coherent, then, to suppose that the reasons why there is a response-time effect in this ball-detection are the very reasons which explain why observing actual actions can accelerate our execution of the same action^9,10^. But of course this could only be true if, as we hypothesise, what observers represent motorically tracks not only the agent’s goals but also her beliefs.

In short, the minimalist design of the ball-detection task can be seen as combining an implicit ToM task with a motor imitation task, thereby providing an almost ideal way to explore Bardi and Brass’s^26^ suggestion that there is a functional connection between belief tracking and action control. Kovacs and colleagues’ discovery about automaticity justifies their postulation of belief tracking being a social sense^30^. If this sense turns out to depend on motor representations of others’ potential actions, we will have discovered that belief tracking is a *bodily* social sense.

**Figure 1:**
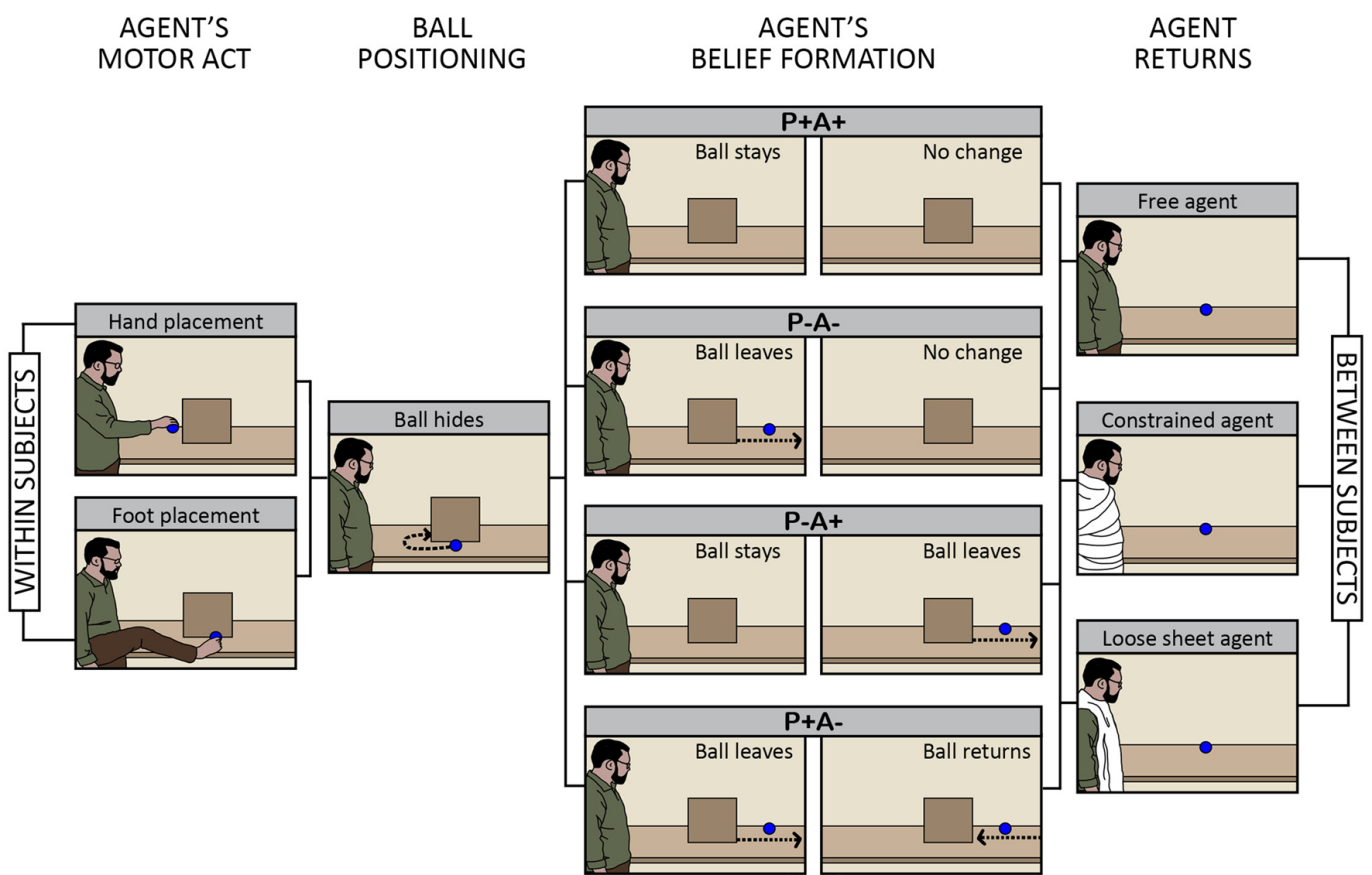
Schematic showing logical structure of the study. There were three versions of the ball-detection task, presenting distinct constraints on the agent’s ability to move and, potentially, to act upon the ball when he returned to the scene (between-subjects manipulation). Participants experienced the Free-Agent version (no sheet), or the Constrained-agent version (sheet restricted potential upper body movement) or the Loose-Sheet version (sheet allowed potential upper body movement). Each group of participants completed two forms of the ball-detection task (within-subject manipulation): in the Hand-Placement form of the task, the agent placed the ball on the table with his hand; in the Foot-Placement form of the task, the agent placed the ball on the table with his foot.

The primary aim of our study was to determine whether and to what extent visibly constraining an agent from potentially interacting with an object modulates observers’ abilities to automatically track that agent’s belief. We measured elicitation of susceptibility to an agent’s belief (as indicated by the P−A+ < P−A− effect) using the ball-detection task. As indicated in Fig. 1, we adapted this task to create three distinct version which differed in the constraints upon the agent’s potential movement to act on the ball in the outcome phase.

In the Free-Agent version of the ball-detection task, the agent returned at the outcome phase in the same clothing; the agent was visibly free to move and, potentially, to act on the ball. In the Constrained-Agent version as shown in Fig. 1, the agent returned at the outcome phase with his upper body and limbs bound up tightly in a white linen sheet (“mummified”); the agent was visibly constrained from using his upper body and limbs to potentially act on the ball. Extrapolating from research evidence showing that physical constraints on others’ action possibilities are mapped onto our own representations of the environment^13,22^, one possibility may be that the effect of the agent’s belief speeding up participants’ own action performances is a consequence of the way that information about beliefs feeds into motor predictions concerning the agent’s potential actions, and the way that those motor predictions would then facilitate response times. With respect to our primary research aim, we predicted that the P−A+ < P−A− effect would be elicited in the free-agent context but obstructed in the constrained-agent context.

One might argue that the obstruction of the critical effect in the Constrained-Agent version as compared to the effect’s elicitation in the Free-Agent version stems from the perceptual novelty of the agent’s (“mummified”) appearance in the former version’s outcome phase. To rule out this low-level possibility, we also studied elicitation of belief tracking in a third version of the ball-detection task. As illustrated in Fig. 1, in the Loose-Sheet version, the agent returned with a white linen sheet (same yardage as in the Constrained-Agent version) placed loosely (like a tunic) over the clothing he wore during the first phase of the video. Consequently, in the Loose-Sheet version, the agent also returned with a novel appearance, but he remained visibly able to move his upper body and limbs and, potentially, to act upon the ball. If perceptual novelty modulates belief tracking, then the P−A+ < P−A− effect should be obstructed in the Loose-Sheet and the Constrained-Agent versions. But if motor representations of action context modulate belief tracking, as per our primary prediction, then the P−A+ < P−A− effect should be elicited in the Free-Agent and Loose-Sheet versions but obstructed in the Agent-Constrained version. The contrast between our predictions over the elicitation of the P−A+ < P−A− effect (in the Free-Agent, Constrained-Agent, and Loose-Sheet versions of the ball-detection task) with the predictions based on perceptual novelty is summarised in Table 1.

**Table 1:** Contrasting predictions about the ball-detection task where visibly constraining an agent from potentially interacting with an object modulates observers’ belief tracking.

Is the P−A + < P−A− effect predicted in this version of the ball-detection task?			
	Free-agent version	Constrained-agent version	Loose-sheet version
Our prediction	Yes	No	Yes
Perceptual novelty prediction	Yes	No	No

A secondary aspect of our study was to learn about whether the P−A+ < P−A− effect might depend on a match between the effector used by the agent to act and the effector used by participants to respond. Considering the hierarchical structure of motor representations wherein information can be instantiated from effector-specific to effector-general levels^16,18^, any indication on whether the P−A+ < P−A− effect depends on matching agent’s and participants’ effectors would bear on whether, if motor representations of action do indeed drive the P−A+ < P−A− effect, those motor representations specify which effector the agent uses to act. Would the effect of belief tracking facilitating participants’ own representations of the environment be elicited only when the agent used his hand to grasp and position the ball? Or might the P−A+ < P−A− effect also be elicited if the agent were to use his foot to interact with the ball? Each of the three versions of the object-detection task (Free-Agent, Constrained-Agent, and Loose-Sheet versions; between-subject manipulation) came in two forms. The Hand-Placement form of the task began with the agent grasping and placing a ball on the table using his right hand; the Foot-Placement form of the task begin with the agent grasping and placing the ball on the table using his right foot (within-subject manipulation and counterbalanced). If motor representations modulating belief tracking are relatively effector-specific, then the P−A+ < P−A− effect should be elicited only, or more strongly, in the Free-Agent and Loose-Sheet versions where the agent had used his hand (rather than foot) to place the ball on the table. If motor representations specify goals but are neutral on the effector used by the agent, then the P−A+ < P−A− effect should be elicited in the Free-Agent and Loose-Sheet versions and elicited regardless of whether the agent had used his hand or his foot to act upon the ball.

## Results

Analysis was undertaken on correct responses on ball-present trials^34^. To address our primary research aim, we performed a 3 (Version: Free-Agent; Constrained-Agent; Loose-Sheet) × 2 (Form: Hand-Placement; Foot-Placement) × 4 (Condition: P+A+; P+A−; P−A+; P−A− mixed model ANOVA. There was only a significant main effect of Condition (*F*(2.734, 366.406) = 113.09, *p* = 0.000, η_p_^2^ = 0.46) that was qualified by a significant interaction effect of Version x Condition (*F*(5.469, 366.406) = 2.58, *p* = 0.022, η_p_^2^ = 0.04). The effector (Hand-placement or Foot Placement) used by the agent to place the ball on the table did not make any difference to participants’ responding; the Form x Version x Condition interaction was not significant (*F*(5.620, 376.568) = 0.70, *p* = 0.641, η_p_^2^ = 0.01). We carried out a one-way ANOVA for each version of the ball-detection task to unpack the significant Version x Condition interaction.

**Figure 2:**
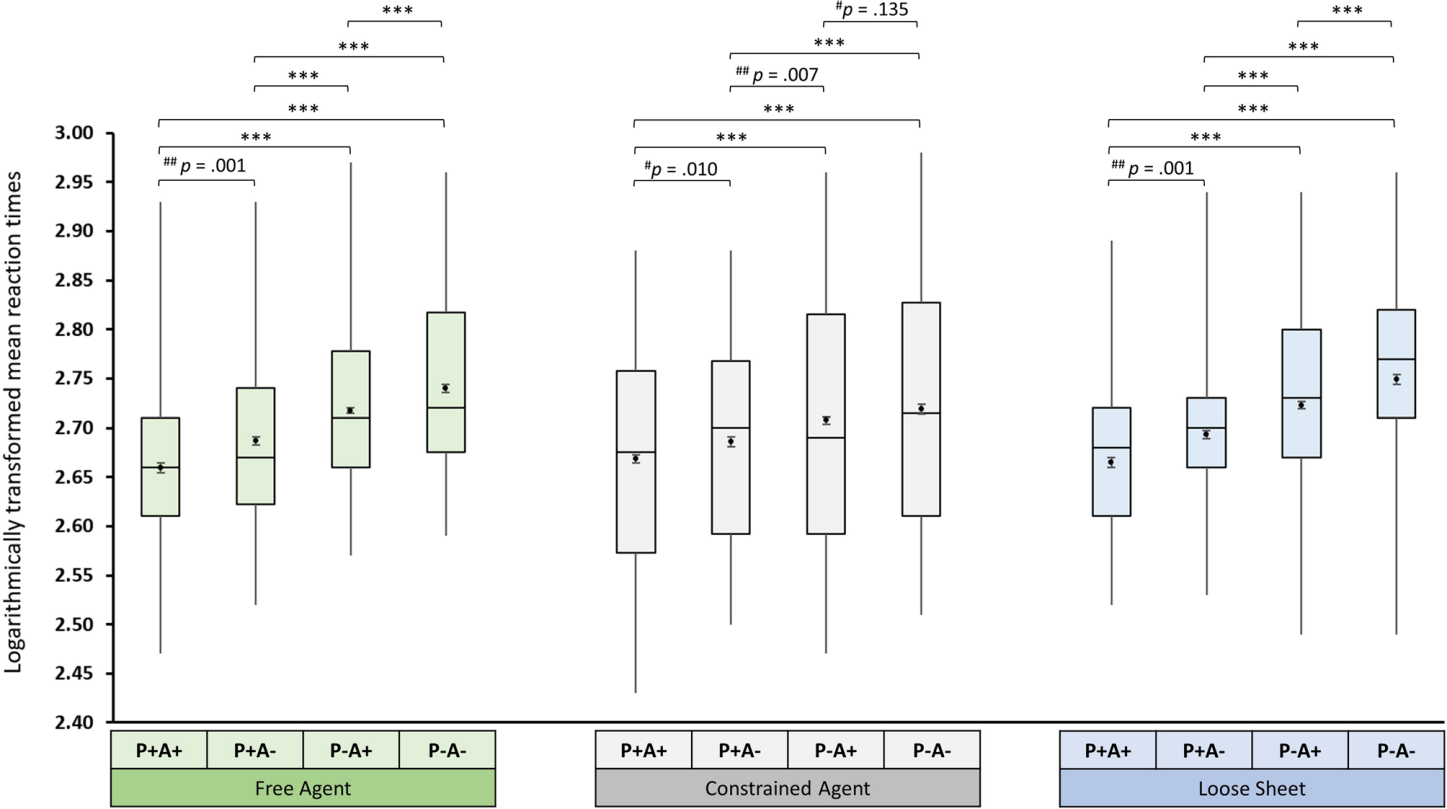
Box plots presenting logarithmically transformed mean response times for the four conditions in each of the different versions of the ball-detection task. Means are represented by dot markers; associated error bars represent the standard error of the mean. The horizontal line in the box represents the median, the length of the box represents the interquartile range, and the whiskers represent the highest and lowest values. Comparisons marked with ***(denoting *p* < .001) and ^##^(denoting *p* < .0083) survived Bonferroni correction for multiple comparisons. Comparisons marked with ^#^(denoting *p* > .0083) did not survived Bonferroni correction for multiple comparisons.

The main effect of Condition was significant in the Free-Agent version (*F*(3, 135) = 54.30, *p* < 0.001, η_p_^2^ = 0.55), in the Constrained-Agent version (*F*(2.62, 117.67) = 20.18, *p* < 0.001, η_p_^2^ = 0.31), and in the Loose-Sheet version (*F*(2.49, 109.41) = 43.09, *p* < 0.001, η_p_^2^ = 0.50). Pairwise t-tests were performed to interpret the significant main effects, with Bonferroni correction (with significance threshold at *p* < 0.0083) to account for multiple comparisons. The pattern of responding is shown in Fig. 2. Supporting our primary prediction, response times were significantly faster in the P−A+ condition than in the P−A− condition in the Free-Agent version (*t*(45) = 4.435, *p* < 0.001; *r* = 0.55) and in the Loose-Sheet version (*t*(44) = 3.796, *p* < 0.001; *r* = 0.50). Also confirming our primary prediction, there was no difference between the P−A+ and P−A− conditions in the Constrained-Agent version (*t*(45) = 1.521, *p* = 0.135; *r* = 0.22).

We then compared response times for the other conditions. The pairwise comparisons of response times for the other conditions are listed in Table 2. The pairwise comparisons indicated that, in all three versions of the ball-detection task, participants were fastest to respond in the P+A+ condition and slowest to respond in the P−A− condition; in addition, their reaction times in the P+A− condition were significantly faster than in the P−A+ condition.

**Table 2:** Overview of pairwise comparisons in each version of the ball-detection task.

Version	Comparison	Paired differences		*t*	*p*
		*m*	*sd*		
Free agent	P−A− versus P−A+	.023	.035	4.435	< .001^a^
	P−A− versus P+A−	.054	.045	8.062	< .001^a^
	P−A− versus P+A+	.081	.051	10.756	< .001^a^
	P−A+ versus P+A−	.031	.045	4.654	< .001^a^
	P−A+ versus P+A+	.059	.047	8.518	< .001^a^
	P+A− versus P+A+	.027	.052	3.561	= .001^a^
Constrained agent	P−A− versus P−A+	.010	.045	1.521	= .135^b^
	P−A− versus P+A−	.032	.055	3.937	< .000^a^
	P−A− versus P+A+	.049	.050	6.743	< .001^a^
	P−A+ versus P+A−	.022	.052	2.839	= .007^a^
	P−A+ versus P+A+	.039	.036	7.418	< .001^a^
	P+A− versus P+A+	.018	.045	2.694	= .010^b^
Loose sheet	P−A− versus P−A+	.026	.046	3.796	< .001^a^
	P−A− versus P+A−	.056	.049	7.645	< .001^a^
	P−A− versus P+A+	.084	.066	8.539	< .001^a^
	P−A+ versus P+A−	.030	.051	3.966	< .001^a^
	P−A+ versus P+A+	.058	.052	7.545	< .001^a^
	P+A− versus P+A+	.028	.050	3.746	= .001^a^

^a^Comparison significant after Bonferroni correction, ^b^comparison did not survive Bonferroni correction.

**Figure 3:**
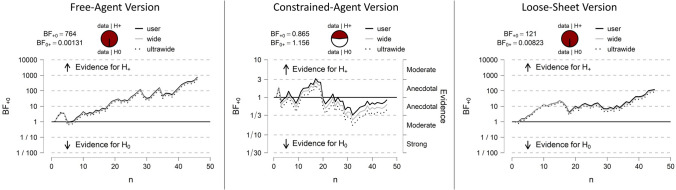
Sequential analysis plots of the Bayes factors for the critical belief-tracking effect (P−A+ < P−A−) in the Free-Agent, Constrained-Agent and Loose-Sheet versions of the ball-detection task.

To complement the traditional (frequentist) methods of statistical analyses of the P−A+ versus P−A− responding in each version of the ball-detection task, we performed Bayesian one-tailed paired t-tests (using JASP 0.12.2 software^40^) to quantify the relative strength of our empirical data for one prediction over another. We used JASP’s default Cauchy prior width^41^ of 0.701 (the software’s own robustness checks confirmed Bayes factors were stable even with wide or ultrawide priors). There was very strong or decisive evidence (by convention^42^, Bayes factors greater than 30) for the P−A+ < P−A− effect in the Free-Agent and Loose-Sheet versions of the ball-detection task. Figure 3 illustrates, by way of the sequential analysis plots provided in the JASP output, the trajectory of the Bayes factors as evidence accumulated. The Bayes factors (BFs) for the Free-Agent and Loose-Sheet versions indicated that the data were 764 times (BF_10_ = 764.21) and 121 times (BF_10_ = 121.474), respectively, more likely under the alternative hypothesis than the null hypothesis. The Bayes factor for the Constrained-Agent version (BF_01_ = 1.16) suggested that that there was anecdotal evidence (by convention, for BFs between 0.33 and 3.00) for slightly favouring the null hypothesis. The pattern of evidence remained the same when Bayesian two-tailed t-tests were performed (Free-Agent version BF_10_ = 382.12; Loose-Sheet version BF_10_ = 60.76; Constrained-Agent version BF_01_ = 2.14). As Bayes factors represent a continuous measure of evidence, we can describe our overall results as suggesting that the P−A+ < P−A− effect was more likely to be shown when the agent was unconstrained and less likely to be shown when the agent was constrained. It is possible that the non-decisive elimination of the P−A+ < P−A− effect in the Constrained-Agent version (BF_01_ = 1.16) suggests belief tracking could still be occurring in the Constrained-Agent version even though it was not modulating participants’ responses. We will take up this possibility in the Discussion section.

Aside from the routine approach of profiling P−A+ versus P−A− responses *within* particular versions of the ball-detection task^30,34^, a few studies have also computed and analysed the resulting difference between the P−A− and P−A+ conditions *across* groups or manipulations^33,36^. We computed and compared participants’ belief-tracking difference scores (i.e., P−A+ subtracted from P−A−) *across* task versions. There was no evidence that the belief-tracking difference score differed across the various versions of the ball-detection task (*F*(2, 134) = 1.84, *p* = 0.162; η_p_^2^ = 0.03) (BF_01_ = 3.00). Dovetailing with our results, some studies have also reported that even though the P−A+ versus P−A− responding may be distinctive at the within-groups level, belief-tracking difference scores may be relatively undifferentiated at the between-groups level^33,36^. We therefore took inspiration from Deschrijver and colleagues’ analytical approach^33^ by performing multiple Levene’s tests (with Bonferroni adjusted p-values at *p* < 0.017) to explore whether the Constrained-Agent version of the task produced more variability than compared to either the Free-Agent or Loose-Sheet versions of the task, but only in the crucial P−A+ condition. In the P−A+ condition, participants in the Constrained-Agent version showed more variance in responding than participants in the Free-Agent version (Levene’s test = 9.89, *p* = 0.002). In the P−A+ condition, participants in the Constrained-Agent version showed more variance in responding than participants in the Loose-Sheet version (Levene’s test = 8.69, *p* = 0.004). In the P−A+ condition, there was no evidence that the variance in responding differed between participants in the Free-Agent version and the Loose-Sheet-version (Levene’s test = 0.02, *p* = 0.891). In the P−A− condition, there was no evidence that the variance in responding differed across versions of the ball-detection task (Bonferroni adjusted *p*-values at *p* < 0.017; *p*s ranged from 0.018 to 0.969). Overall, it appears that constraining the agent in the ball-detection task introduced more variability in response times in the crucial P−A+ condition. This variability may reflect unreliability in participants’ belief tracking in the Constrained-Agent condition, which would explain why we found only anecdotal evidence against the presence of an P−A− versus P−A+ effect in the Constrained-Agent version, and why we did not find evidence that the belief-tracking score differed across the various versions of the ball-detection task.

We also undertook an orthogonal analysis of the ball-present data to explore the influence of the agent’s belief and the participant’s belief^34^. We performed a 3 (Version: Free-Agent; Constrained-Agent; Loose-Sheet) × 2 (Form: Hand-Placement; Foot-Placement) × 2 (Belief Holder: P, A) × 2 (Belief: +, −) mixed model ANOVA. We computed four scores, P+, P−, A+ and A−. The A+ score captured the scenario where the agent was led to expect the outcome ([P+A+] + [P−A+]/2), whereas the A-score captured the scenario where the outcome was unexpected by the agent ([P+A−] + [P−A−]/2). The P+ score captured the scenario where the participant expected the outcome (([P+A+] + [P+A−])/2), whereas the P-score captured the scenario where the participant did not expect the outcome (([P−A+] + [P−A−])/2). The mixed model ANOVA revealed two significant interactions, which are explained below.

The significant main effect of Belief Holder (*F*(1, 134) = 20.10, *p* < 0.001; η_p_^2^ = 0.13) was qualified by a significant Belief Holder x Belief interaction (*F*(1, 134) = 43.26. *p* < 0.001; η_p_^2^ = 0.24). The interaction can be explained by the observation that the effect of belief (+ or −) was stronger when it was the participants themselves that held the mental state than when it was the agent that held the mental state. In order words, the difference between P + (*M* = 2.68, *SD* = 0.09) and P− (*M* = 2.73, *SD* = 0.11) (*t*(136) = 14.38, *p* < 0.001; *r* = 0.79) responding was larger than that between A + (*M* = 2.69, *SD* = 0.10) and A− (*M* = 2.71, *SD* = 0.10) (*t*(136) = 7.07, *p* < 0.001, *r* = 0.52) responding.

The significant main effect of Belief (*F*(1, 134) = 218.63, *p* < 0.001; η_p_^2^ = 0.62) was qualified by a significant Belief x Version interaction (*F*(2, 134) = 4.74, *p* = 0.010; η_p_^2^ = 0.07). The interaction can be explained as follows. The difference between a ball-expected-absent (−) and a ball-expected-present (+) belief on task responding was larger in the Free-Agent version (*M*_Difference_ = 0.04, *SD* = 0.03) than compared to the Constrained-Agent version (*M*_Difference_ = 0.03, *SD* = 0.03) ((*t*(90) = 2.81, *p* = 0.006; *r* = 0.28). Similarly, the difference between a ball-expected-absent (−) and a ball-expected-present (+) belief on task responding was larger in the Loose-Sheet version (*M*_Difference_ = 0.04, *SD* = 0.03) than compared to the Constrained-Agent version ((*t*(82.73) = 2.64, *p* = 0.010; *r* = 0.28). There was no evidence of a difference in belief effect on task responding between the Loose-Sheet and Free-Agent versions ((*t*(82.33) = 0.24, *p* = 0.809, *r* = 0.03). There was also no evidence that the Belief x Version interaction was due to the differing effect of the agent’s belief across versions (that is, no Version x Belief-Holder x Belief interaction); although we did not predict any such effect, finding one would have lent additional support to our hypothesis about mummification impairing participant’s abilities to manifest belief tracking.

Finally, we checked that participants showed a high level of accuracy (regardless of either the version or form of the ball-detection task), revealed by low mean error proportions across the different trial conditions. Tests for normality revealed that the error data was positively skewed. Pooling across the different versions, a Friedman test revealed no statistically significant differences in mean error proportions across the four trial conditions (P+A+ = 0.06; P+A− = 0.07; P−A+ = 0.07; P−A− = 0.07; Χ^2^(3) = 5.95, *p* = 0.114).

## Discussion

We hypothesised that information about beliefs can feed into motor predictions concerning an observed agent’s potential actions. To test this hypothesis, we adapted a widely replicated ball-detection false belief task. Given existing evidence that such motor predictions can accelerate our execution of the same action and decelerate our execution of an incompatible action^9,10^, our hypothesis generates the prediction that these motor predictions may be responsible for the P−A+ < P−A− (false-belief tracking) effect observed in the ball-detection task. To test this prediction, we used “mummification” to manipulate whether the observed agent in our ball-detection task was free to act (Free-Agent version) or whether he was visibly constrained from acting (Constrained-Agent version). We found evidence for the P−A+ <P−A− effect in the Free-Agent version but not in the Constrained-Agent version, and evidence indicating that participants’ responses times in the Constrained-Agent version did not reliably reflect the agent’s belief.

Our findings converged with studies of interference effects showing that physical constraints on others’ action possibilities are mapped onto observers’ own representations of the environment^13,22^. Costantini et al.^13^ documented that reaction-time advantages in adults’ predispositions to act towards a graspable mug—triggered whenever participants observed that the target object was presented within another person’s reaching space—disappeared when a transparent barrier was interposed between the agent and the mug. Their argument was that the transparent barrier prevented any potential action on the part of the agent, and inhibition of motor stimulation impacted upon participants’ own representation of appropriate goal-related acts towards the object. Our findings demonstrate that reaction-time effects which are sensitive to an observed agent’s beliefs (and not merely to her action possibilities) can be similarly modulated by preventing any potential action on the part of the observed agent.

Just here we face an objection. Participants may not have shown reaction-time benefits in the Constrained-Agent version of the ball-detection task because the agent’s “mummified” appearance in the outcome phase was perceptually novel. Anticipating this objection, we included a third, Loose-Sheet, version of the ball-detection task as a control for perceptual novelty. In the Loose-Sheet version, the agent also returned with a novel appearance involving a sheet, but he remained visibly able, potentially, to act upon the ball. We found that the P−A+ < P−A− effect was elicited in the Free-Agent *and* Loose-Sheet versions. This is in line with the predictions of our hypothesis and contrary to what we would expect if the objection about perceptual novelty were correct.

The inspiration for our research was Bardi and Brass’s^26^ suggestion that motor processes and belief tracking may be connected. It is now well established that motor processes may be influenced by facts about the goals of unseen or withheld actions^13,43,44^. The stimuli in the ball-detection task also withheld from showing the agent reaching for a ball behind the wall at the outcome phase. Yet, as we hypothesised, whether or not the agent was able to potentially act on the ball turned out to be critical for the realisation of false-belief tracking. This finding raises the new and exciting possibility that motor representations in an observer may support tracking not merely an observed agent’s goals but also their beliefs.

Theoretically, this opens the door to many exciting possibilities. One such possibility is that automatic belief-tracking processes are distinct from, but can influence, motor processes. If this possibility is correct, we would expect that belief tracking is still occurring in the Constrained-Agent version of the ball-detection task, but it is not reliably feeding into motor processes to modulate participants’ responses. Another, more radical, possibility is that some automatic belief-tracking processes are so closely bound up with motor processes that impairing the motor processes also impairs the belief-tracking processes. If so, we would expect that belief tracking is not occurring (or not occurring in the same way) in the Constrained-Agent version.

More research will be needed to determine whether belief-tracking processes occur even when motor processes in participants are impaired. For example, aside from measuring the latencies of participants’ key-press responses, researchers might simultaneously measure skin conductance and pupil dilation effects^45–47^. If automatic belief-tracking processes are motor processes, we would expect “mummification” to eliminate indications of belief tracking not only in response times but also in skin conductance and pupil dilation. A further question is whether impairing motor processes could modulate nonautomatic belief-tracking processes. To address this question, our “mummification” technique could be used in classic false-belief tasks^48^.

An important limit of our modified ball-detection task is that the agent’s physical constraints, as shown in Fig. 1, appear only after the ‘Agent’s Belief Formation’ stage. If belief-tracking processes already occur in the ‘Agent’s Belief Formation’ stage of our scenario (see Fig. 1), then the radical idea that these processes can not only influence motor processes but may also depend on them predicts that belief tracking would also be impaired if the agent were constrained during the agent’s belief-formation phase. Our mummification technique could be extended to test this further prediction.

Do our findings allow us to predict that wherever we have a fast belief-tracking process, impairing motor representations will impair the process? We think they do not. Our findings indicate that *some* of the fast belief-tracking processes may influence, or may even be bound up with, motor processes. But accepting this conclusion leaves us open to the possibility that *other* fast belief-tracking processes may have nothing to do with motor processes. For instance, some belief-tracking may be based on perceptual processes. We therefore remain open to the idea that there is heterogeneity in the processes and representations supporting belief tracking.

A secondary aspect of our study was to learn about whether the false-belief tracking effect could depend on there being a match between the effector used by the agent to act and the effector used by participants to respond. The form of the effector (hand or foot) used by the agent to place the ball on the table did not make any difference to the latencies of participants’ key-press responses. It is possible that the hand-placement versus foot-placement occurred too early in the event sequence for the manipulation to make much difference to participants’ detection of the ball. It is also possible that with a different paradigm, it may turn out that belief tracking might be effector specific. That said, there are studies supporting the idea that there may be hierarchical levels of processing that go beyond specifying elementary motor features such as patterns of joint displacements or muscle contractions; some motor activations can selectively discharge according to the anticipated outcome to which an action is potentially directed towards, regardless of the specific effector used^16,18^. With respect to the present findings, our cautious interpretation is that we regard response times on the ball-detection task as reflecting a sort of motor process that is influenced by belief tracking, but the motor process is unlikely to mainly take into account the specific effector involved.

Our findings bear on a broader issue that concerns the situatedness by which observers would need to distinguish motor representations that are self-triggered and other-triggered. This issue arises given that witnessing someone else’s action activates corresponding motor representations in observers. In some circumstances, failures to distinguish self- and other-triggered motor representations can have acute functional consequences. For instance, patients with Parkinson’s disease may find it challenging to impose their own internally planned movements against externally perceived movements, which may lead to over-imitation and postural instability^49^. What we have shown is that failures to do so are not always dysfunctional: without the possibility for others’ potential actions and beliefs to influence our motor representations we would lack an embodied social sense.

To sum up, we have been building on evidence about the role of motor processes in enabling us to understand others. Until now, most evidence concerns tracking the goals of others’ actions. Our novel finding is that manifesting false-belief tracking abilities can be a consequence of the way that mental state information influences our motor predictions of others’ potential actions, and the way that those motor predictions then facilitate our reactions. Our findings signal that motor processes may underpin the primary ways in which human beings engage in social cognition.

## Methods

### Participants

A total of 144 adult participants, made available by the Victoria University of Wellington’s Introduction to Psychology Research Programme, signed up to take part in the study. Of these, 48 participants were randomly allocated to each of three versions of the object-detection task. An a priori analysis using G*Power^50^ (input parameters: α = 0.05, power = 0.8) determined that a sample size of at least 33 participants was required to detect the standardised effect size in each version. While not a direct replication of Kovács et al.^30^, our sample size was based on the standardised effect size of their critical effect (*r* = 0.45). This was calculated using the formula, *r*^2^ = *t*^2^/(*t*^2^ + *df*), where *t* = reported t-test statistic of Kovács et al.’s critical effect = 2.42, and *df* = 23. Having a larger number of individuals safeguarded against participant dropout, and other factors affecting data collection such as experimenter error or computer malfunction. Of the 48 participants originally allocated to the Free-Agent version, one participant was removed due to a technical fault and one participant did not perform above a 75% accuracy threshold. As a result, analysis was undertaken on the data of 46 participants (mean age, 18.76 years; range, 17–22; male to female ratio, 10/36). Of the 48 participants allotted to the Constrained-Agent version, two participants did not perform above a 75% accuracy threshold meaning that analysis was undertaken on the data of 46 participants (mean age, 18.98 years; range, 18–29; male to female ratio, 13/33). Of the 48 participants allotted to the Loose-Sheet version, three participants did not perform above a 75% accuracy threshold so that analysis was undertaken on the data of 45 participants (mean age, 19.29 years; range, 17–44; male to female ratio, 10/35). In total, analysis was undertaken on the data of 137 participants. The ratio of females to males was 104/33 and the mean age was 19.01 years (range 17 to 44).

All methods were carried out in accordance with relevant guidelines and regulations. Experimental protocols were approved by Victoria University of Wellington’s School of Psychology under delegated authority of the Victoria University of Wellington Human Ethics Committee. All participants signed informed consent forms prior to participation and were debriefed orally at the end of the session.

### Materials

**Figure 4:**
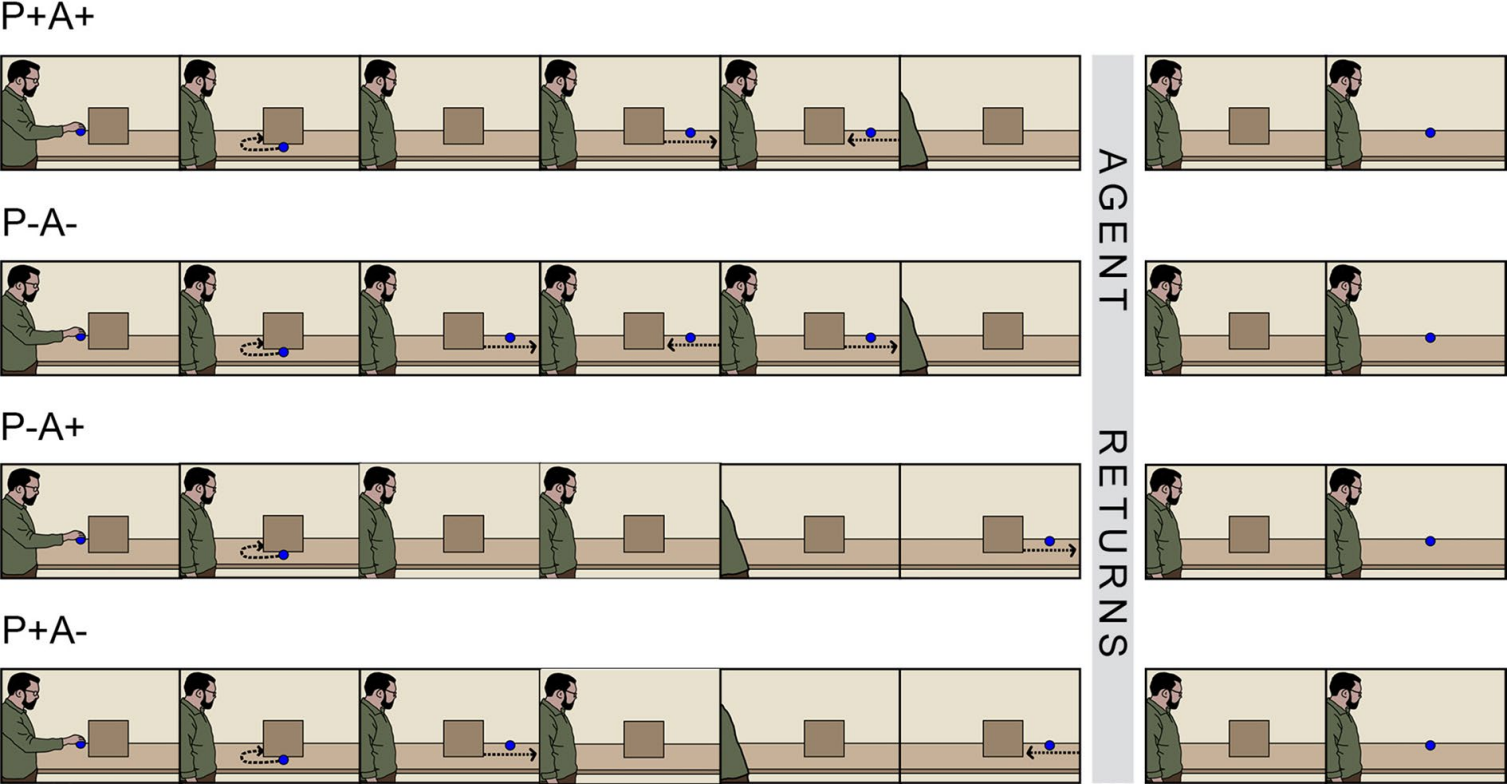
Schematic showing main events of the four belief-inducing conditions in the Constrained-Agent version (ball present trials). In the P+A+ condition, both participant and agent expect the ball to be present; in the P−A− condition neither participant nor agent expect the ball to be present. In the P+A− condition only the participant expects the ball to be present, and in the P−A+ condition only the agent expects the ball to be present. The events for the Loose-Sheet and Free-Agent versions were identical except that in the former the agent returned with a sheet loosely draped over his upper body, and in the latter the agent returns with no sheet (appearance unchanged).

We used E-Prime 2.0 for the experiment. Participants in each of the three versions of the ball-detection task watched a total of 80 videos. The videos were 38 cm x 21 cm for on-screen presentation and were 21 s in length (25 frames per second with 720 × 576 resolution). Participants experienced two forms of their respective version of the object-detection task (adapted from Edwards & Low^34^); these forms differed according to whether the agent placed an object on a table with his hand or his foot (order counterbalanced). There were 40 videos involving hand placements and 40 videos involving foot placements. Given that the primary aim of our study was to discover whether and to what extent visibly constraining an agent from potentially interacting with an object modulates observers’ abilities to automatically track that agent’s belief, we shall illustrate events in the videos of the ball-detection task by way of the Constrained-Agent version. The events in the different conditions of the Constrained-Agent version of the ball-detection task are shown in Fig. 4.

### Constrained-Agent version

For the Constrained-Agent version involving hand placements, each video began with an agent (a real human actor) standing in front of a table with a wooden wall. The agent held a ball in his outstretched hand. The agent placed the ball in front of the wall and a series of events ensued in which the ball moved around the table in a self-propelled manner. In the ball’s first movement, it moved behind the wall so that it could not be seen by either the participant or agent. Following this movement, the events in the videos varied to create four belief-induction conditions.

The belief-induction conditions differed according to whether the participant expected a ball to be present (P+) or absent (P−) in the outcome phase, and whether the agent expected a ball to be present (A+) or absent (A−) in the outcome phase. In adapting Edwards and Low’s^34^ stimuli, we briefly outlined here the four belief-inducing conditions when paired with a ball present outcome. In the P+A+ condition, both participant and agent were led to believe there was a ball behind the wall. In the P−A− condition, neither the participant nor the agent was led to believe that there was a ball behind the wall. In the P+A− condition, the participant, but not the agent, was led to believe the ball was behind the wall. In the P−A+ condition, only the agent had reason to believe that there was a ball behind the wall.

In the outcome phase, the agent returned to the same location as at the start of the video, but the agent was tightly bound in a white linen sheet (i.e., “mummified”) which restricted his upper body and hand movements. His presence signalled the final event of the video which depicted one of two outcomes: 1) when the wall rapidly fell away, the ball was present (ball-present outcome); or 2) when the wall rapidly fell away, the ball was not present (ball-absent outcome). As such, participants experienced 8 trial types, comprised of four belief-induction conditions paired with one of two possible outcomes.

The videos showing foot placements by the agent were identical to those showing hand placements by the agent, except that each video commenced with the agent placing the ball on the table with his right foot rather than his right hand.

### Free-Agent version

The Free-Agent version (whether the agent used his hand or his foot to place the ball on the table) were identical to those of the Constrained-Agent version except that the agent returned to the scene in the same guise and he had his arms free by his sides.

### Loose-Sheet version

The Loose-Sheet version (whether the agent used his hand or his foot to place the ball on the table) were identical to those of the constrained-Agent version except that when the agent returned to the scene in the outcome phase, a loose white linen sheet hung over his shoulders so that arm and hand movements were unrestricted.

### Procedure

Participants were tested individually at a Dell Optiplex 9,020 desktop (23″ screen with 16:9 aspect ratio). Following Edwards and Low^34^, the initial screen stated: “This is an object-detection task. Your job is to press a key as quickly as you can when you see something appear behind a wall”. Participants then completed the Hand-Placement and Foot-Placement forms of the task (counterbalanced). The instructions, based on Edwards and Low^34^, for both forms were identical except for the information in brackets: “In the first (second) half of the experiment you will see 40 videos, lasting a total of about 15 min. They will look like this (relevant frame of video provided). In each video, the person will leave the scene, then return. Press the ‘Q’ key with your left hand as soon as the person has completely left the scene. When the wall disappears do one of the following with your right hand: Press the ‘N’ key if the ball is present; Press the ‘M’ key if the ball is absent”.

In both forms of the said version of the ball-detection task, each trial consisted of an initial fixation cross (1000 ms), followed by a brief video. The following procedures were also based on Edwards and Low^34^. We required participant to make two responses for each video: an attention check (pressing a key within 2000 ms of the agent leaving the scene), and an object detection (selecting whether or not the ball was revealed when the wall dropped away). The timings of each trial’s events were the same across tasks (see Figure S1 of Supplementary File for event timings). For each form, 40 test trials were presented in a pseudorandom order in two blocks. The first block contained 24 trials comprising three cycles of four different conditions with a ball-present or ball-absent outcome. After a student-led break the participants experienced another block of 16 trials (two cycles of four different belief-inducing conditions with either a ball-present or ball-absent outcome). The participants were then instructed to undertake a second round of the ball-detection task. Thus, across the hand placement and foot placement forms of the task, participants experienced 80 trials in total.

At the start of each form of the task, a training phase exposed the participants to 4 practice trials with response time feedback. No performance feedback was given during the test phase to minimise trial time and distraction. The entire experiment took approximately 35 min in total. Following Edwards and Low’s^34^ procedure, on completion of the experiment participants were asked to complete a form purportedly surveying their experience of how easy it was to sign up for laboratory experiments in exchange for partial course credits (e.g., “Have you found it easy to find suitable timeslots?”). The final question, “What was the experimenter testing?” sought to determine whether the participants were primed to consider the bystander’s belief. Although not a funnelled debriefing protocol we were confident from survey answers that mental state attribution was not deemed to be the target of our research; all participants’ answers referred to the measuring of attention or reaction times in the pursuit of object detection.

### Data processing and analysis

Analysis was undertaken on correct responses^32,34^, defined as those in which the participant accurately detected the presence of the ball (readers interested in pairwise comparisons involving ball-absent data of conditions in each version may refer to Table S1 of the Supplementary File). All traditional statistical tests were two-tailed^34^. Reaction times for trials in which participants failed to respond to an attention check were excluded (1.5% of trials in the Free-Agent version, 2.3% of trials in the Constrained-Agent version, and 2.7% of trials in the Loose-Sheet version). Following Edwards and Low’s^34^ approach to outlier analysis, all data points greater than 3 standard deviations above or below the participant’s overall mean in each task were removed. As a result, 63 individual RTs were omitted from the Free-Agent version (1.7% of total), 51 from the Constrained-Agent version (1.4% of total), and 60 from the Loose-Sheet version (1.7% of total). Tests for normality revealed a positive skew in reaction times and error rates. A logarithmic transformation of the reaction time data was performed to fit the assumptions of an ANOVA before proceeding with further statistical analyses. As such, where means are reported in the main text, the means describe logarithmically transformed data. The extent of the positive skew for the error data necessitated non-parametric testing. Greenhouse Geisser corrections were used whenever the assumption of sphericity was violated.

## Supplementary information

Supplementary file1 (XLSX 40 kb)

Supplementary file2 (PDF 259 kb)
