# Supplementary material for: Visibly constraining an agent modulates observers’ automatic false-belief tracking
Source: Sci Rep. 2020 Jul 9;10:11311. doi: 10.1038/s41598-020-68240-7 (PMC7347931; doi:10.1038/s41598-020-68240-7)
Supplement: Supplementary file 2 — Supplementary file2 (PDF 259 kb) [file 41598_2020_68240_MOESM2_ESM.pdf]

Visibly constraining an agent modulates observers' automatic false-belief tracking (Appendix/Supplementary Figure and Table)

Jason Low[1*], Katheryn Edwards[2], Stephen A. Butterfill[3]

[1]School of Psychology, Victoria University of Wellington, Wellington 6140, NZ.
[2]School of Sport, Health and Wellbeing, Plymouth Marjon University, Plymouth PL6 8BH, UK.
[3]Department of Philosophy, University of Warwick, Warwick CV4 7AL, UK.

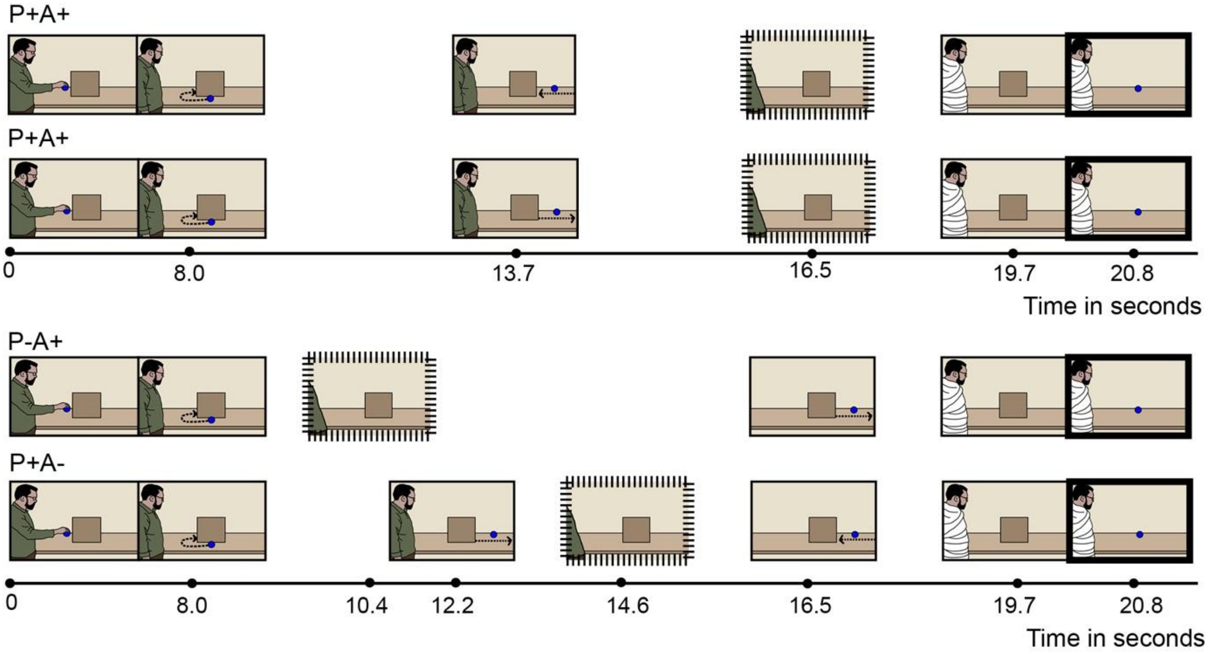

Figure S1. *Timings of the main events of the four conditions (illustrated by way of the Constrained-Agent version of the ball-detection task) (timing are the same in the other task versions). Two main events are highlighted: (1) the attention check triggered by the exit of the agent (hatched border); (2) removal of the screens (bold border).*

Table S1. *Overview of pairwise comparisons in each version of the ball-detection task for ball-absent trials.*

		Paired Differences			
**Version**	**Comparison**	***m***	***sd***	***t***	***p***
**Free Agent**	P−A− *versus* P−A+	.016	.049	2.206	=.033[b]
	P−A− *versus* P+A−	-.037	.053	-4.744	<.001[a]
	P−A− *versus* P+A+	-.049	.059	-5.635	<.001[a]
	P−A+ *versus* P+A−	-.053	.054	-6.639	<.001[a]
	P−A+ *versus* P+A+	-.065	.062	-7.212	<.001[a]
	P+A− *versus* P+A+	-.012	.041	-2.011	=.050[b]
**Constrained Agent**	P−A− *versus* P−A+	.007	.037	1.291	=.203[b]
	P−A− *versus* P+A−	-.031	.052	-3.985	<.000[a]
	P−A− *versus* P+A+	-.041	.062	-4.465	<.001[a]
	P−A+ *versus* P+A−	-.038	.057	-4.488	<.001[a]
	P−A+ *versus* P+A+	-.048	.064	-5.100	<.001[a]
	P+A− *versus* P+A+	-.010	.051	-1.389	=.172[b]
**Loose Sheet**	P−A− *versus* P−A+	.016	.061	1.721	=.092[b]
	P−A− *versus* P+A−	-.041	.075	-3.615	=.001[a]
	P−A− *versus* P+A+	-.051	.067	-5.108	<.001[a]
	P−A+ *versus* P+A−	-.056	.068	-5.583	<.001[a]
	P−A+ *versus* P+A+	-.066	.064	-6.926	<.001[a]
	P+A− *versus* P+A+	-.010	.045	-1.531	=.133[b]

Note: [a]*effect remained significant after Bonferroni correction;* [b]*effect did not survive Bonferroni correction.*
